# Advances in the Management of Rh Haemolytic Disease

**Published:** 1966-07

**Authors:** Geoffrey H. Tovey

**Affiliations:** Medical Director, South West Regional Transfusion Service Clinical Teacher in Haematology, University of Bristol


### ADVANCES IN THE MANAGEMENT OF Rh HAEMOLYTIC DISEASE

GEOFFREY H. TOVEY, M.D., F.C. PATH.

*Medical Director, South West Regional Transfusion Service*
*Clinical Teacher in Haematology, University of Bristol*

Exchange transfusion of the newly born baby affected with Rh hæmolytic disease was introduced by Diamond in 1947. As a result of its fuller development, and particularly repeated exchange transfusion for severe cases, at least 95 per cent of live-born affected babies may be saved by this treatment (Walker, 1959). Attention is now being directed therefore to the prevention of stillbirth, and, more recently, to the eventual elimination of the disease by protecting the Rh negative mother against the formation of Rh antibodies.

### PREVENTION OF STILLBIRTH

Rh hæmolytic disease is still too widely regarded as a condition in which the risk of stillbirth is relatively slight and then only after one or more mildly or moderately affected babies. However, the established facts are that about 1 in 20 Rh negative mothers develops antibodies during her second and third pregnancy; that 8-10 per cent of these first-affected babies are so severely affected that they will die *in utero* unless special measures are taken; and that once severe disease has appeared in the family nearly all subsequent fœtuses, unless they are Rh negative, are likely to be more severely affected. As Zuelzer and his colleagues (1965) have recently remarked the old adage " once a stillbirth, always a stillbirth " is a salutary warning in this condition.

In their analysis of 183 stillbirths from Rh incompatibility, Zuelzer *et al.* found that 107 (or 75%) occurred in the first pregnancy in which the mother developed antibodies, and it is clear that, until measures can be taken to prevent Rh iso-immunisation, the principal attack must be to prevent stillbirth in first-affected pregnancies. Tovey and Valaes (1959) have established that death *in utero* is almost confined in such pregnancies to those in which the mother develops antibodies in high titre; by their technique this titre is 1/20 to 1/40. They showed also that approximately two-thirds of such stillbirths occurred after the 36th week of the pregnancy. As a result, it has since 1959 become the practice in Bristol to induce delivery at 36-37 weeks if the titre of anti-Rh reaches 1/40 before the 36th week. This practice has more recently been adopted in Bath and Taunton, and an analysis of 659 first-affected pregnancies has enabled an assessment to be made of the outcome of this change in policy (Table I).

TABLE I

Rh hæmolytic disease in first-affected pregnancies: Comparative figures 1955-61 and 1962-64.

| | 1955-61 (Bristol Area) | 1962-64 (Bristol and N. Somerset) |
|---|---|---|
| Total cases ... ... | 307 | 352 |
| Perinatal loss ... ... | 27 (8.8%) | 19 (5.4%) |
| S.B. after 36 weeks ... | 7 | 2 |
| Neonatal deaths ... ... | 6 | 1 |

Premature delivery based on antibody titre was already in practice towards the end of the period 1955-61, and without this the loss by stillbirth after the 36th week would have been even higher. In the period 1962-64, during which induction became more generally applied, stillbirth after 36 weeks almost disappeared and the over-all perinatal loss was reduced from 8.8 to 5.4 per cent. Of the two stillbirths which occurred after the 36th week, one was at full term and the mother had not reported for an antibody test until she was 39 weeks pregnant.

During 1962-64, twelve of the 352 first-affected fœtuses were stillborn between the 34th and 36th weeks of the pregnancy (Table II). From this analysis it is evident that when an Rh negative mother develops a high titre of Rh antibodies there is a 10 per cent risk that she will lose her baby by stillbirth about four to six weeks before full term. Unfortunately, prediction of severe disease by antibody titre alone is not sufficiently precise to detect affected babies and enable some at least to be saved by even earlier delivery. Although most babies are severely affected when there is a high antibody titre, at a titre of 1/40 one in seven babies is so mildly affected that it does not require exchange transfusion. The hazards of prematurity outweigh therefore the possible benefits to be obtained by delivery before 36 weeks when prediction has to be based only on the antibody titre.

TABLE II

Time of stillbirth related to antibody titre in 352 first-affected pregnancies.

| Titre | Number of Cases | Stillbirth after 36 weeks | Stillbirth at 34-36 weeks | Stillbirth before 43 weeks |
|---|---|---|---|---|
| Below 1/20 ... ... | 240 | 0 | 0 | 0 |
| At or above 1/20 ... | 112 | 2 (1.8%) | 12 (10.5%) | 4 (3.5%) |

### AMNIOTIC FLUID ANALYSIS

Destruction of fœtal red cells *in utero* results in the accumulation of bilirubin-like pigments in the amniotic fluid. Prediction of severity based on amniotic fluid analysis was pioneered by Bevis (1956) and subsequently developed by Walker and Jennison (1962) and Liley (1961). A preliminary report of the value of this procedure in the management of hæmolytic disease in the South West Region was given in this Journal in 1965 by Branch.

The method used at Southmead Hospital (Holton, 1966) is that developed by Liley in which, following the spectrophotometric measurement of the increased optical density of the amniotic fluid at 450 nm, the result is plotted on a logarithmic scale divided into three zones. The upper zone indicates severe red cell destruction with a high incidence of stillbirths; the lower zone indicates an unaffected or mildly affected baby; and the middle zone indicates, generally, a moderately affected baby. Our own experience of 102 cases has confirmed that although predicition is possible with considerable accuracy in the upper and lower zones, it cannot be made with the same degree of confidence when the pigment value falls within the middle zone.

TABLE III

Amniotic fluid analysis in 102 cases of Rh hæmolytic disease.

| Zone | Total Babies | Correctly Predicted | More Severely Affected | Less Severely Affected | Fœtal Loss |
|---|---|---|---|---|---|
| Upper ... | 22 | 22 | 0 | 0 | 15 (70%) |
| Middle ... | 60 | 46 | 5* | 9† | 2 (3.3%) |
| Lower ... | 20 | 20 | 0 | 0 | 0 |

* 2 hydrops † 6 Rh negative

Despite this limitation, amniotic fluid analysis affords a major advance towards predicting more accurately which fœtus may die *in utero*. Already the lives of three severely diseased first-affected babies have been saved in the South West Region following this method of prediction. In each case a high antibody titre at 32-34 weeks called attention to the need for amniocentesis; the amniotic pigment values found in the three fluids were in the upper zone; in two of the babies born at 34 weeks the cord blood hæmoglobins were only 4 g. per 100 ml. and in the third, born at 35 weeks, 4.5 g. per 100 ml. All three babies made a good recovery following repeated exchange transfusions.

Determination of antibody titre is now being re-directed therefore towards deciding whether and when amniocentesis is required. During the period 1962-64 none of the 352 first-affected babies reviewed in Table II was stillborn when the antibody titre remained below 1/20. Amniocentesis is recommended therefore only if the titre reaches 1/20 before the 36th week of the pregnancy. As a result about one in three mothers requires amniotic fluid analysis during a first affected pregnancy (Table II). Amniocentesis should not be carried out without proper indication because of the risk of placental hæmorrhage and premature labour. The accidental entry of fœtal red cells into the mother's circulation through placental damage may lead to further immunisation of the mother with a sharp rise in antibody titre and a more severely affected baby at the present or a subsequent pregnancy.

### INTRA-UTERINE TRANSFUSION

In 1963 Liley reported the first successful transfusion of the fœtus *in utero*, having made use of the fact that red blood corpuscles are absorbed intact into the circulation from the fœtal peritoneal cavity, and that absorption is complete within a period of three or four days. This technique is now being used in an attempt to prevent stillbirth when the amniotic fluid analysis gives an upper zone pigment value and the fœtus is too immature to be saved by immediate delivery. Under premedication and local anæsthesia, a plastic or Teflon catheter is introduced into the baby's peritoneal cavity through a 16 cm. needle. Modern radiographic equipment, incorporating image intensification and T.V. monitoring, greatly facilitates the procedure and, when satisfactory insertion has been confirmed by injecting 1-2 ml. of a water-soluble radio-opaque medium, 100-160 ml. of group 0 Rh negative concentrated red cells are injected slowly into the peritoneal cavity. If necessary, the transfusion is repeated every 10-12 days until the fœtus is at least 35-36 weeks mature.

Of 52 desperate cases recently reported (Green, Liley and Liggins, 1965; Karnicki, 1966), 24 babies (46 per cent) were born alive and saved by subsequent exchange transfusion. In 21 of the 28 unsuccessful cases the fœtus was already hydropic at the time of the attempted intra-uterine transfusion, and it is concluded therefore that transfused red cells are not absorbed sufficiently rapidly from the peritoneal cavity to save the fœtus when ascites is present.

### PRESENT DAY MANAGEMENT OF RHESUS-SENSITISED PREGNANCIES

It has been customary to examine blood samples from Rh negative mothers for antibodies at the 34th week of the pregnancy. If early stillbirths from hæmolytic disease are to be prevented it is now clear that these tests must be brought forward. The following procedure is therefore recommended:

(1) The first sample (5-10 ml. of clotted blood) should be taken at 8-12 weeks, or as soon as the patient is known to be pregnant. The routine syphilis tests are carried out on this sample and it is "screened" for Rh antibodies. This sample should be sent in every pregnancy, even if the mother is Rh positive, in case immune antibodies have developed following the previous pregnancy.

(2) If no antibodies are detected in the first sample and the patient is (a) Rh negative and multiparous, or (b) has received a blood transfusion at any time (whether she is Rh negative or Rh positive) a second sample should be taken at 28 weeks for antibody tests.

(3) If no antibodies are found in this sample, a third one should be tested at 34 weeks.

When Rh antibodies are found, their titre is determined, and if it is a first-affected pregnancy, amniocentesis will be indicated if the titre reaches 1/20. If the antibody titre is 1/80 or more at the 28th week, amniocentesis should be performed without delay, otherwise the optimum time will be at 31-32 weeks.

When the amniotic pigment value is found to be in the upper zone, intra-uterine transfusion will have to be considered, unless the pregnancy is already at 34 weeks gestation and the fœtus is sufficiently mature to have a good chance of survival following premature delivery. When the pigment value lies in the middle zone between 30-34 weeks, a further examination should be made within 2-3 weeks to determine whether the pigment is rising into the upper zone, indicating that the fœtus is becoming more severely affected.

For women who have already carried an affected baby, the antibody titre should be determined at about the third month of the pregnancy. The timing of an amniocentesis will then depend upon the extent to which the previous baby was affected, and upon the antibody titre. If there is a history of stillbirth before the 36th week, amniotic fluid examination is desirable at 24 weeks, since intra-uterine transfusion may have to be performed as early as 26-28 weeks if hydrops and death *in utero* are to be prevented. Amniocentesis is advisable whenever there has been a previously affected baby, unless the baby was so mildly affected that it did not require treatment and the antibody titre is no higher in the current pregnancy. Except when the history is bad or the antibody titre is high, amniocentesis is best delayed until 31-32 weeks so as to avoid repeated examinations.

Pre-term delivery is undesirable when the fœtus is only mildly affected, since it adds the possible jaundice of immaturity to that of hæmolytic disease. When the amniotic pigment value is in the lower zone, or in the case of a first-affected baby the antibody titre remains below 1/20, the pregnancy should be allowed to continue to, but not beyond, full term.

### PREVENTION OF RHESUS SENSITISATION

As long ago as 1944 Levine reported that ABO blood group incompatibility between the fœtus and its mother appeared to prevent the Rh negative mother from developing

Rh antibodies. Race and Sanger (1950) suggested that because group A or B Rh positive fœtal red cells would be rapidly destroyed by the mother's naturally occurring anti-A or anti-B antibodies they would be less likely to immunise the mother and cause her to develop Rh antibodies than would group O (compatible) fœtal cells if they entered her circulation. They concluded that compatible fœtal cells would remain sufficiently long in the mother's blood to reach tissue sites responsible for antibody stimulation. These theoretical considerations have been brilliantly put to clinical test by research teams in Liverpool (Clarke *et al.*, 1966) and New York (Gorman *et al.*, 1965), and their results to date provide great hope that it may soon prove possible to prevent Rh negative women from developing Rh antibodies.

It has been established that the major cause of sensitisation of the mother to the Rh antigen is a fœto-maternal transfusion of ABO compatible Rh positive fœtal red cells during labour (Finn *et al.*, 1961). In Liverpool and New York therefore attempts are being made to prevent sensitisation by detecting fœtal bleeds, and destroying any Rh positive fœtal cells in the mother's blood by injecting a high concentration of anti-Rh in the form of gammaglobulin as soon as her baby has been delivered. The gamma-globulin must not be injected before delivery because it may, of itself, cause hæmolytic disease in an otherwise healthy fœtus. The results of this trial to date are given in Table IV.

TABLE IV

Current results of trial injections of gamma globulin for the prevention of iso-immunisation to the Rh antigen.

| | Developed Antibodies | No antibodies |
|---|---|---|
| Given gamma globulin ... | 0 | 78 |
| Controls : not injected ... | 21 | 73 |

These results are most encouraging, but it has been agreed that this procedure will not be more generally applied until some of the mothers included in this trial have had a further pregnancy in which the fœtus is Rh positive, to establish that prevention of sensitisation has been complete. The possibility that carrying a subsequent Rh positive fœtus may still stimulate Rh antibodies early in the pregnancy must first be excluded. Should it prove possible, as seems very likely, that Rh hæmolytic disease may be prevented by injecting gammaglobulin whenever an Rh negative mother is delivered of an unaffected Rh positive baby, the blood transfusion and obstetric services will be faced with a formidable task in finding and processing sufficient anti-Rh in the form of gammaglobulin and then of injecting every Rh negative mother who is delivered of an ABO compatible Rh positive baby. To be effective, it is probable that the injection will have to be given not later than 48 hours after delivery.

It must be noted that the injection of gammaglobulin is of no value when the mother has already formed Rh antibodies. It cannot inhibit such antibodies or prevent them being further stimulated to a high titre during pregnancy. Even, therefore, when hæmolytic disease becomes preventable, it is unfortunately likely to be many years before there will no longer be need to predict severe disease in the fœtus, and to provide facilities for intra-uterine and exchange transfusion.

I wish to thank my obstetric and pædiatric colleagues in Bristol, Bath and Taunton for their co-operation in the investigation and management of cases of hæmolytic disease in their clinical areas; Dr. J. B. Holton and his staff for the amniotic fluid examinations; and the laboratory staff of the Regional Transfusion Centre for assistance with the Rh serology and antibody titres.

### REFERENCES

Bevis, D. C. A. (1956). *J. Obstet. Gynaec. Brit. Comm.*, **63**, 68.

Branch, R. A. (1965). *Bristol Med.-Chir. J.* **80**, 61.

Clarke, C. A. *et al.* (1966). *Brit. Med. J.* **1**, 213.

Diamond, L. K. (1947). *Proc. Roy. Soc. Med.* **40**, 546.

Finn, R. *et al* (1961). *Brit. Med. J.* **1**, 1486.

Gorman, J. G., Freda, V. J. and Pollack, W. (1965). *Lancet,* **2**, 181.

Green, G. H., Liley, A. W. and Liggins, G. C. (1965). *Aust. New Zeal. J. Obstet. and Gynaec.* **5**, 53.

Holton, J. B. (1966). Personal communication.

Karnicki, J. (1966). *Nursing Mirror,* 7th Jan., P.1.

Levine, P. (1944). *Amer. Arch. Path.*, **37**, 83.

Liley, A. W. (1961). *Amer. J. Obstet. Gynaec.* **82**, 1359.

Liley, A. W. (1963). *Brit. Med. J.* **2**, 1107.

Race, R. R. and Sanger, R. (1950). Blood Groups in Man. 1st Edn. Blackwell, Oxford.

Tovey, G. H. and Valaes, T. (1959). *Lancet* **2**, 521.

Walker, A. H. C. and Jennison, R. F. (1962). *Brit. Med. J.* **2**, 1152.

Walker, W. (1959). *Brit. Med. Bull.*, **15**, 123.

Zuelzer, W. *et al* (1965). *Amer. J. Obstet. Gynaec.* **92**, 627.

